# Development of a Closed One-Tube Colorimetric and Fluorescent LAMP Assay for the Rapid Detection of Feline Herpesvirus 1

**DOI:** 10.3390/ani16111651

**Published:** 2026-05-28

**Authors:** Shushuai Yi, Han Zhao, Xinying Huang, Jiaxin Yu, Wanli Sha, Wei Lian, Jiangting Niu, Baishuang Yin

**Affiliations:** 1 College of Animal Science and Technology, Jilin Agricultural Science and Technology College, Jilin 132101, China; yishushuai888@jlnku.edu.cn (S.Y.); zhaohan4566@163.com (H.Z.); huangxinyingsyx@163.com (X.H.); mrsuanwork@163.com (J.Y.); jlshawanli@163.com (W.S.); 2 Jilin Zhengye Biological Products Co., Ltd., Jilin 132101, China; zyyanfa123@163.com

**Keywords:** feline herpesvirus 1, one-tube colorimetric and fluorescent LAMP, cresol red, visual detection, clinical application

## Abstract

Feline herpesvirus type 1 (FHV-1) is one of the major pathogens causing upper respiratory tract disease (URTD) in cats, and is widely prevalent among domestic cats and other felids worldwide. Herein, we developed a closed one-tube colorimetric and fluorescent LAMP assay targeting the FHV-1 TK gene, using cresol red as the colorimetric indicator and EvaGreen as the fluorescent dye. The assay was completed within 40 min at 63 °C, with results readable either by colorimetric observation or fluorescence amplification curve. FHV-1-positive reaction could be confirmed by a color change from violet to yellow with the naked eye, and also could be determined by fluorescence amplification curve. The assay showed no cross-reactivity with other related pathogens, and had a detection limit of 100 copies. Among 87 clinical nasal swabs tested, 44 were positive, achieving an overall agreement of 97.7% compared to TaqMan quantitative PCR. The newly developed LAMP assay is simple, efficient, result-visual and reliable, which can be applied in the clinical diagnosis and epidemiological investigation of FHV-1, especially for point-of-care testing of FHV-1 in primary veterinary clinics and field settings.

## Simple Summary

Feline herpesvirus type 1 poses a serious threat to the health of domestic cats. It is important for the clinical diagnosis of FHV-1 to develop a point-of-care testing (POCT) method. In this study, a closed one-tube colorimetric and fluorescent LAMP assay was developed innovatively using cresol red as the colorimetric indicator and EvaGreen as the fluorescent dye. This is the first report of a colorimetric and fluorescent dual-mode LAMP assay applied for the detection of FHV-1. This assay offers the following advantages: (1) closed-tube detection prevents aerosol contamination; (2) integration of visual colorimetric detection and real-time fluorescence quantification; (3) rapid detection, with results obtainable within 40 min; (4) high specificity; (5) a detection limit of 100 copies by visual colorimetric detection; and (6) excellent reproducibility and stability, making it well-suited for POCT of FHV-1.

## 1. Introduction

Feline upper respiratory tract disease (URTD), also known as feline respiratory disease complex (FRDC), is one of the most common and significant infectious diseases affecting domestic cats worldwide. Feline URTD poses a severe challenge in multi-cat environments such as animal shelters, rescue centers, and breeding catteries, where it is a leading cause of morbidity and a primary reason for euthanasia [1]. Feline URTD is a multifactorial syndrome caused by diverse pathogens, including feline herpesvirus-1 (FHV-1), feline calicivirus (FCV), *Chlamydia felis* (*C. felis*), *Mycoplasma felis* (*M. felis*), *Bordetella bronchiseptica* (*Bb*), *Streptococcus pneumoniae* (*Sp*), etc. [1,2]. Co-infections occur in 20–50% of cases, leading to more severe clinical disease. Furthermore, these pathogens cause nearly identical clinical symptoms, such as sneezing, ocular and nasal discharge, conjunctivitis, etc., making it difficult to differentiate diagnosis.

FHV-1 belongs to the family *Herpesviridae*, subfamily *Alphaherpesvirinae*, and genus *Varicellovirus* [3], and is an enveloped, double-stranded DNA virus. The viral particles have a diameter of approximately 120–180 nm. The FHV-1 genome is approximately 136 kb in length, comprising unique long (UL), unique short (US), terminal repeat sequences (TRS) and internal repeat sequences (IRS) regions [4]. The FHV-1 genome contains approximately 78 open reading frames (ORFs), encoding 74 proteins, among which gB, gD, and TK proteins are highly conserved and essential for infectivity and pathogenicity [5], making them ideal diagnostic targets. FHV-1 is primarily transmitted through direct or indirect contact with respiratory or ocular secretions, with 2–4-month-old kittens being most susceptible. Acute infection causes upper respiratory symptoms such as conjunctivitis, keratitis, sneezing, and serous to purulent nasal and ocular discharge [6]. Severe cases lead to pneumonia and secondary bacterial infections, with up to 50% mortality in kittens [6]. Survivors develop lifelong latent infection in the trigeminal ganglia, and viral reactivation causes recurrent disease, complicating control [7]. Recently, FHV-1 infections have been reported in wild felids, including leopard cats [8], cheetahs [9,10], pumas [11], jaguars [12], and the critically endangered South China tigers [13], posing significant challenges to the conservation of these wild species. Therefore, early, rapid and specific detection is crucial for the prevention and control of FHV-1 infection.

Currently, polymerase chain reaction (PCR) and quantitative PCR (qPCR) have been widely used in the clinical diagnosis of FHV-1 [14,15,16]. However, these techniques are time-consuming, operationally complex, and require specialized personnel and instrumentation, making them unsuitable for point-of-care testing (POCT) in primary animal hospital and field settings. Loop-mediated isothermal amplification (LAMP), first reported by Notomi et al. in 2000 [17], enables exponential nucleic acid amplification at a constant temperature using *Bst* DNA polymerase’s strand-displacement and polymerase activity [18]. LAMP products can be detected via colorimetry, turbidimetry, lateral flow dipsticks (LFD), and fluorescence [19]. The assay is simple, rapid, highly specific, and sensitive, with visually interpretable results, which has been widely applied in POCT for many pathogens, such as SARS-CoV-2 [20], Dengue virus [21], African swine fever virus (ASFV) [22], and feline coronavirus (FCoV) [23], etc.

In this study, we developed a closed one-tube colorimetric and fluorescent LAMP assay for the rapid detection of FHV-1, using cresol red as the colorimetric indicator and EvaGreen as the fluorescent indicator. We designed a specific LAMP primer set targeting the conserved TK gene, optimized the reaction conditions, and then evaluated the specificity, sensitivity, and repeatability. This cost-effective and user-friendly LAMP assay is especially suitable for POCT in animal hospitals and field settings, facilitating timely diagnosis and control of FHV-1 infection.

## 2. Materials and Methods

### 2.1. Viruses, Bacteria and Clinical Samples

FHV-1 isolate CH-B, FCV isolate CH-JL2, feline panleukopenia virus (FPV) isolate CC-02/16, Pseudorabies virus (PRV) attenuated vaccine strain Bartha-K61, *Bordetella bronchiseptica* (*Bb*) and *Streptococcus pneumoniae* (*Sp*) were all maintained at −80 °C in our laboratory. *C. felis*-, *M. felis*- and Canine herpesvirus 1 (CHV-1)-positive nasal discharges and feline coronavirus (FCoV)-positive ascites were confirmed through qPCR and sequencing analysis, and kept in our laboratory. A total of 87 nasal swabs were collected from suspected feline URTD cases from four animal hospitals in Jilin province, China. All nasal swabs were resuspended with 500 μL phosphate-buffered saline (PBS) and centrifuged at 10,000× *g* for 10 min. Then, the supernatant was stored at −80 °C.

### 2.2. Nucleic Acid Extraction

Viral DNA and RNA were extracted using FastPure Viral DNA/RNA Mini Kit (Vazyme, Nanjing, China) according to the manufacturer’s instructions. Total DNA of *C. felis*, *M. felis*, *Bb* and *Sp* were extracted using FastPure Microbiome DNA Isolation Kit (Vazyme, Nanjing, China). The concentration of extracted DNA/RNA was detected using a NanoDrop spectrometer (Thermo Scientific, Wilmington, NC, USA).

### 2.3. Primers Design

TK gene (GenBank accession no. FJ478159) was used to design the suitable LAMP primer sets using the online software Primer explorer version 5 (https://primerexplorer.eiken.co.jp/lampv5e/index.html (accessed on 14 March 2025)) based on oligonucleotide length, GC content and thermodynamic stability. Four sets of primers were designed in our study, each set of primers included two outer primers (B3 and F3), two inner primers (FIP and BIP), and two loop primers (LF and LB). The oligonucleotide sequences of primer sets were listed in Table S1, and all primers were synthesized by Comate Bioscience Co., Ltd. (Jilin, China).

### 2.4. Construction of pMD-TK Standard Plasmid

Primer pairs TK-F/TK-R (oligonucleotide sequences showed in Table 1) were designed to amplify the complete TK gene of FHV-1. The PCR amplification was performed using viral DNA of FHV-1 isolate CH-B as template in a total volume of 50 μL, which contained 10 μL of 5 × TransStart FastPfu Buffer, 4 μL of dNTP mixture (2.5 mM), 1 μL of each TK-F and TK-R (10 μM), 1 μL of FastPfu DNA Polymerase (TransGen, Beijing, China), 3 μL of template DNA and 30 μL of nuclease-free water. The reaction procedure was as follows: predenaturation at 95 °C for 5 min, followed by 35 cycles of denaturation at 95 °C for 30 s, annealing at 58 °C for 30 s, extension at 72 °C for 45 s, and final extension at 72 °C for 7 min. PCR products were electrophoresed in 1% agarose gels and visualized under UV light (Figure S1). Amplicons were cloned into pGEM-T vector to yield positive standard plasmid pMD-TK. The concentration of pMD-TK plasmid was measured using a NanoDrop spectrometer (Thermo Scientific, USA), while the DNA copies were calculated using an online DNA Copy Number and Dilution Calculator (https://www.thermofisher.cn).

### 2.5. LAMP Reaction and Primer Set Selection

The one-tube closed LAMP assay for FHV-1-specific detection was developed using cresol red as the colorimetric indicator and EvaGreen as the fluorescent indicator. To screen for the most suitable primer set, four sets of primers were evaluated using LAMP. The LAMP was performed in a 25 μL of reaction mixture, which contained 2.5 μL of 10 × isothermal amplification buffer (200 mM Tris-HCl, 100 mM (NH_4_)_2_SO_4_, 1.5 M KCl, 20 mM MgSO_4_, 1% Tween-20, pH 8.8), 1.5 μL of MgSO_4_ (100 mM), 3.5 μL of dNTPs (10 mM), 2.5 μL of 10 × LAMP primer mixture (2 μM each of F3 and B3, 16 μM each of FIP and BIP, and 4 μM each of LF and LB), 1 μL of *Bst* 3.0 polymerase (8000 U/mL, New England Biolabs, Lpseich, MA, USA), 0.5 μL of cresol red (0.65 mM), 0.5 μL of 50 × EvaGreen dye (Biotium, Fremont, CA, USA), 1 μL of template DNA and 12 μL of nuclease-free water. FHV-1 isolate CH-B was used as a positive template, while nuclease-free water as a negative control throughout the experiments. All amplifications were repeated three times. The reaction was incubated using a LightCycler^®^ 96 (Roche, Basel, Switzerland) at 65 °C for 45 min, followed by terminated reaction at 80 °C for 5 min. Fluorescence signals were collected every 30 s in the SYBR Green channel. The LAMP reaction results were determined using the following procedures: (a) fluorescence amplification curve generated by LightCycler^®^ 96. The time to positivity (Tp) was determined when the LAMP reactions achieved a fluorescence threshold calculated based on the fluorescence values of the negative control over the initial 15 cycles. (b) Colorimetric observation with the naked eye. Positive reactions showed a change in color from violet to yellow, while negative reactions remained violet. (c) Electrophoresis analysis using 2% agarose gel. The characteristic ladder-like bands were observed in positive reactions, but not in negative reactions.

### 2.6. Optimization of the LAMP Assay

To achieve the optimal performance of the LAMP assay, the following essential parameters were optimized sequentially using a one-variable-at-a-time (OVAT) method: amplification temperature ranging from 59 °C to 65 °C with 1 °C gradient interval, concentration of inner primers with inner-to-outer primer (keeping the same final concentration at 0.2 μM) ratio ranging from 2:1 to 9:1 (the final concentration of inner primer was 0.4 to 1.8 μM with 0.2 μM gradient interval), concentration of loop primers with loop-to-outer primer (keeping the same final concentration at 0.2 μM) ratio ranging from 1:1 to 6:1 (the final concentration of loop primer was 0.2 to 1.2 μM with 0.2 μM gradient interval), final concentration of Mg^2+^ (including that from isothermal amplification buffer and the added MgSO_4_) ranging from 3 to 8 mM with 1 mM gradient interval, concentration of dNTPs ranging from 0.8 to 1.8 mM with 0.2 mM gradient interval, and reaction time ranging from 10 to 60 min with 10 min gradient interval. The optimal conditions were determined based on the Tp value, endpoint fluorescence and the change in color.

### 2.7. Specificity Analysis

To assess the specificity, total DNA and RNA extracted from FCV, FPV, FCoV, CHV-1, PRV, Bb, Sp, C.felis and M.felis were tested under the optimal reaction conditions. FHV-1 isolate CH-B and pMD-TK plasmid were used as positive control, while nuclease-free water was a negative control.

### 2.8. Comparative Sensitivity of LAMP and qPCR

The pMD-TK plasmid was 10-fold serially diluted using nuclease-free water with a variation in concentration from 10^0^ copy/μL to 10^7^ copies/μL. A total of 1 μL of serially diluted plasmid was detected using the developed LAMP assay to determine the limit of detection (LOD). To compare the sensitivity of the LAMP assay, one qPCR method (primer sequences listed in Table 1) developed by Cao et al. [14] was also used to detect the serially diluted plasmids. Briefly, the qPCR was performed using Taq Pro HS Probe Master Mix (Vazyme, China) by adding 0.4 μL each of forward and reverse primers (10 μM), 0.2 μL of probe primer (10 μM), 1 μL of template plasmid and 8 μL of nuclease-free water to 10 μL of 2 × Taq Pro HS Probe Master Mix. The reaction was carried out using the following procedure: predenaturation at 95 °C for 30 s, followed by 40 cycles of 95 °C for 10 s and 60 °C for 30 s with fluorescence acquisition.

### 2.9. Repeatability Analysis

To evaluate the repeatability of the LAMP assay, three diluted positive plasmids with the concentration of 10^2^ copies/μL, 10^4^ copies/μL and 10^6^ copies/μL were tested using the developed LAMP assay. The experiment repeated three times for each concentration.

### 2.10. Validation in Clinical Samples

A total of 87 clinical samples were detected by the developed LAMP assay and qPCR for FHV-1. 3 μL of nucleic acid extracted from clinical samples was used for both assays. The coincident rate, comparative sensitivity and comparative specificity of the LAMP assay was calculated using a two-by-two table based on qPCR as the reference assay. The *kappa* coefficient value was analyzed using the Chi-square test in SPSS 18.0 software. The interpretation of the *kappa* value refers to the previous description [24].

### 2.11. Heat Map Analysis

The real-time fluorescence amplification curves of the LAMP assay were visualized as heat maps using GraphPad Prism 8.2.0 software. The rainbow color indicates the progression from low to high fluorescence intensities, while white color indicates that the fluorescence intensity is lower than the fluorescence threshold.

## 3. Results

### 3.1. Screening of Suitable Primer Set

**Figure 1:**
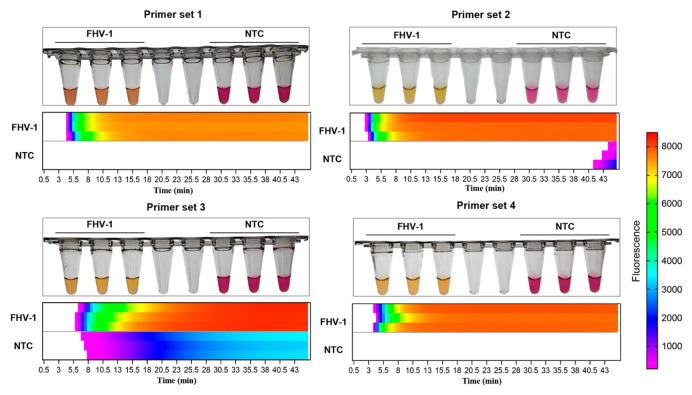
The selection of the optimal primer set of the closed one-tube colorimetric and fluorescent LAMP assay. Different primer sets (set 1–4) were used to amplify FHV-1 DNA (FHV-1) and nuclease-free water (NTC). Upper rows: colorimetric observation; positive reactions turned yellow, while negative remained violet. Lower rows: fluorescence amplification curve; amplification is represented as the normalized fluorescence, shown in a colorimetric scale. All experiments were performed in triplicate.

To select the most suitable primer set, four sets of primers were tested under the typical LAMP protocol. The suitable primer set was determined based on the shortest Tp value, highest endpoint fluorescence value, brightest color change, ladder-like bands and non-specific amplification. As shown in Figure 1, all four sets of primers induced a specific violet-to-yellow color change with FHV-1 DNA, while negative controls showed no color change. However, non-specific fluorescence curves appeared in negative controls for primer sets 2 and 3. Between primer sets 1 and 4, no significant Tp difference was observed, but set 4 produced higher endpoint fluorescence, more intense color change, and the brightest ladder-like bands on agarose gel electrophoresis (Figure S2). The oligonucleotide sequences of primer set 4 and their location within the FHV-1 TK gene are listed in Table 1.

### 3.2. Optimum Reaction Conditions of the LAMP Assay

**Figure 2:**
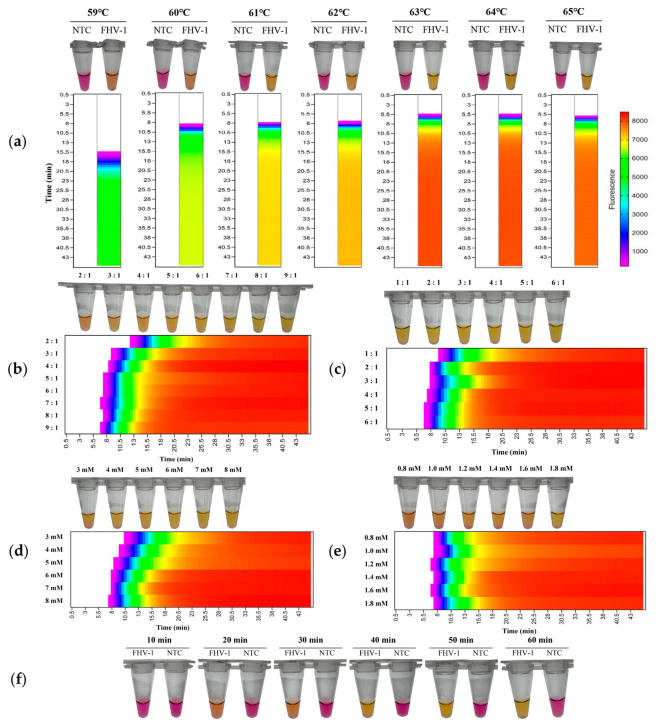
Optimization of the closed one-tube colorimetric and fluorescent LAMP assay for FHV-1 detection. (**a**) Reaction temperature; (**b**) inner primer concentration (ratio of inner to outer primers is listed in figure); (**c**) loop primer concentration (ratio of loop to outer primers is listed in figure); (**d**) final Mg^2+^ concentration; (**e**) dNTPs concentration; (**f**) amplification times. Upper rows: colorimetric observation; yellow color indicates positive reaction and violet color indicates negative reaction. Lower rows: fluorescence amplification curve; amplification is represented as the normalized fluorescence, shown in a colorimetric scale.

To determine the optimal reaction conditions for the LAMP assay, six parameters were optimized sequentially in this study. For amplification temperature, positive reactions exhibited a clear violet-to-yellow color change between 59 °C and 65 °C, with the most intense yellow at 63–65 °C. Fluorescence amplification curves confirmed the lower Tp values and higher endpoint fluorescence in this temperature range, with no significant differences (Figure 2a). Thus, 63–65 °C was determined as the optimal amplification temperature range. With the outer primer concentrations fixed at 0.2 μM, other primer concentrations were optimized. As seen in Figure 2b,c, the lowest Tp value, highest endpoint fluorescence and brightest yellow color were observed in a 7:1 ratio of inner to outer primers and a 5:1 ratio of loop to outer primers. The optimal primer concentrations were determined to be as follows: 0.2 μM for each outer primer, 1.4 μM for each inner primer, and 1.0 μM for each loop primer. Subsequent optimizations showed that the optimal Mg^2+^ final concentration was 6 mM (Figure 2d) and the optimal dNTP concentration was 1.6 mM (Figure 2e). Using 10,000-fold-diluted FHV-1 DNA as a template, positive color changes were observed after 20–60 min incubation, but not at 10 min. The color intensity appeared brighter between 40 and 60 min, with no significant difference (Figure 2f). An amplification time of 40 min was determined for the developed LAMP assay.

The optimal reaction system contained 1 × isothermal amplification buffer (2 mM MgSO_4_), an additional 4 mM MgSO_4_, 1.6 mM dNTPs, 1 × LAMP primer mixture (0.2 μM each of F3 and B3, 1.4 μM each of FIP and BIP, and 1.0 μM each of LF and LB), 8 U *Bst* 3.0 polymerase, 13 μM cresol red, and 1 × EvaGreen dye, incubated at 63 °C for 40 min.

### 3.3. Specificity of the LAMP Assay

**Figure 3:**
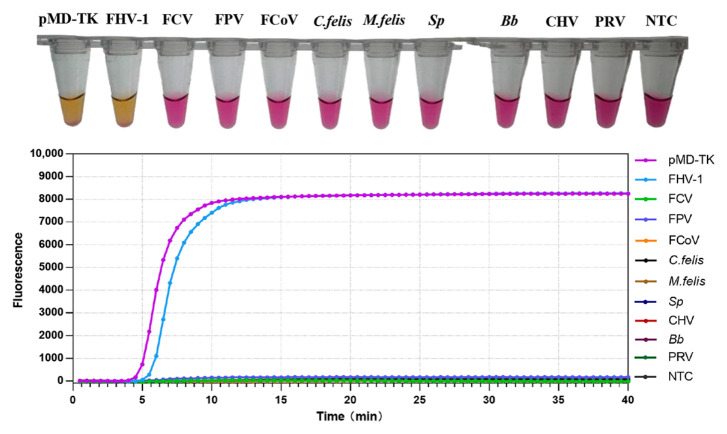
Specificity of the closed one-tube colorimetric and fluorescent LAMP assay. Colorimetric observation shown in upper rows; yellow color indicates positive reaction and violet color indicates negative reaction. Fluorescence amplification curve shown in lower rows. NTC, negative control.

The specificity of the LAMP assay was evaluated against other common pathogens causing feline URTD, other common feline viruses, as well as PRV and CHV-1 (both belonging to the family *Herpesviridae*). As shown in Figure 3 and Figure S3, only FHV-1 isolate CH-B and pMD-TK plasmid showed yellow color and fluorescence amplification curves, while other testing pathogens remained violet in color and exhibited no fluorescence amplification curves exceeding the threshold, identical to the negative control. The results indicated that the developed LAMP assay shows no cross-reactions among the tested pathogens, including FCV, FPV, FCoV, CHV-1, PRV, *Bb*, *Sp*, *C. felis* and *M. felis*, demonstrating high specificity for FHV-1 detection.

### 3.4. Sensitivity of the LAMP Assay

**Figure 4:**
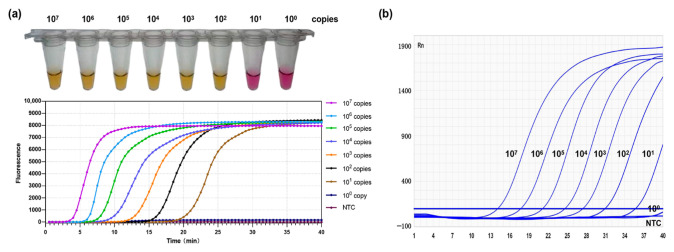
Comparative sensitivity between the closed one-tube colorimetric and fluorescent LAMP assay (**a**) and qPCR assay (**b**). Colorimetric observation shown in upper rows; yellow color indicates positive reaction and violet color indicates negative reaction. Fluorescence amplification curve shown in lower rows. NTC, negative control.

The 10-fold serial dilutions of the plasmid pMD-TK were used to determine the sensitivity of the developed LAMP assay. The qPCR assay served as a reference assay to compare the sensitivity. The LAMP assay could detect plasmid pMD-TK as low as 100 copies via visual colorimetric observation, while it achieved a detection limit of 10 copies when monitored through real-time fluorescence amplification curves (as shown in Figure 4a and Figure S4). The qPCR assay was able to detect plasmid pMD-TK as low as 10 copies (Figure 4b). The results indicated that the developed LAMP assay achieved a comparable limit of detection (LOD) to qPCR when monitored via fluorescence amplification curves, while the visual colorimetric detection exhibited a 10-fold-higher LOD compared to the qPCR assay.

### 3.5. Repeatability of the LAMP Assay

**Figure 5:**
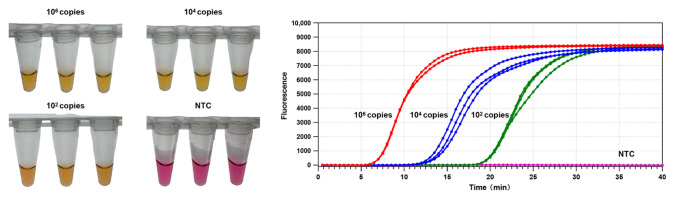
Repeatability of the closed one-tube colorimetric and fluorescent LAMP assay. Colorimetric observation shown in left rows; yellow color indicates positive reaction and violet color indicates negative reaction. Fluorescence amplification curve shown in right rows. NTC, negative control.

Three dilutions of plasmid pMD-TK were used as templates to evaluate the repeatability and stability of the LAMP assay through intra-assay and inter-assay experiments. As shown in Figure 5, the violet-to-yellow color change was consistently observed across repeated tests for each plasmid concentration. As shown in Table 2, the coefficients of variation (CV%) for the Tp values in both intra-assay and inter-assay experiments were below 2% and 5% across all concentrations, suggesting that the developed LAMP assay exhibits a high degree of stability and repeatability.

### 3.6. Clinical Sample Testing and Comparison with qPCR

To confirm the applicability of the developed LAMP assay for the detection of clinical samples, a total of 87 nasal swabs were determined. The tested results of the LAMP assay were compared to those of the qPCR assay. Among the 87 tested samples, 44 samples were identified as positive through colorimetric observation and fluorescence amplification curves, consistent with the qPCR assay. Two samples of negative results with the LAMP assay produced positive results with a relatively high cycle threshold (Ct) value when detected using qPCR assay. Compared to qPCR, the LAMP assay demonstrated a comparative sensitivity of 95.7% (with 95% confidence interval (CI): 82.9–99.2%) and a comparative specificity of 100% (95% CI: 89.9–100.0%), with an overall agreement of 97.7% (Table 3). The kappa coefficient (k) value was 0.95, indicating a substantial level of agreement.

## 4. Discussion

FHV-1 is a primary etiological agent of feline URTD, accounting for 20–50% of clinical cases globally [6,7]. FHV-1 infections pose substantial threats to the conservation of domestic and wild felids. Consequently, there is an urgent need to develop rapid and reliable diagnostic methods to enhance clinical detection and epidemiological surveillance of FHV-1. Isothermal amplification techniques (IATs), characterized by operational simplicity, time-saving, results visualization, sensitivity and specificity, have emerged as promising alternatives to PCR-based methods for nucleic acid detection [25]. IATs are considered highly suitable for point-of-care testing (POCT) for infectious disease. Among IATs, LAMP has been widely adopted for the detection of pathogens due to its high specificity, cost-effectiveness, diverse testing methods, and the availability of well-established primer design software.

The diversity of detection methods for amplification products is a major advantage of LAMP assay. The final amplification products of LAMP consist of a mixture of stem–loop DNA structures with varying sizes, as well as multi-branched, cauliflower-like DNA structures. These products can be separated by agarose gel electrophoresis, which typically reveals ladder-like bands—a characteristic indicator of successful amplification [18,19]. Furthermore, LAMP can be detected based on byproducts generated during amplification, including the double-stranded DNA accumulation, Mg^2+^ changes, or pH shifts. Various dyes enable visual or instrumental detection: fluorescent intercalating dyes, metal ion indicators, and pH-sensitive dyes [19]. Among these dyes, the color changes induced by fluorescent dyes and Calcein require observation under ultraviolet or blue-light excitation [19]. The color changes from violet to sky-blue produced by hydroxynaphthol blue (HNB) may be difficult to distinguish with the naked eye [26]. In contrast, pH-sensitive indicators produce pronounced and easily distinguishable color transitions, making them particularly suitable for colorimetric LAMP detection. Cresol red, which changes from violet to yellow with minimal pH shifts, has been used for the LAMP assay development against SARS-CoV-2 [27], hepatitis A virus [28], porcine circovirus type 3 [29] and *Staphylococcus aureus* [30]. Therefore, cresol red was selected as the colorimetric indicator for this study. EvaGreen dye was also incorporated to enable real-time quantitative detection, expanding the assay’s utility for viral load monitoring.

The highly conserved TK gene of FHV-1 was selected as the diagnostic target, consistent with multiple detection methods, including conventional PCR, TaqMan qPCR, nanoPCR, RPA, and RAA [14,15,16,31]. Four sets of primers targeting the TK gene were designed and screened for optimal performance. Two loop primers (LF and LB) were added to accelerate reaction kinetics, reducing amplification time by 1/3 to 1/2 [32]. Furthermore, both the reaction system and temperature are critical for optimizing the amplification efficiency of LAMP. For instance, the Mg^2+^ concentration can influence the amplification rate, primer concentration can influence the primer–dimer formation and amplification efficiency, incubation temperature can influence the amplification specificity and sensitivity, etc. [18,19]. Consequently, critical reaction parameters were systematically optimized in this study. The final assay operates at 63 °C for 40 min, with results interpretable either visually via cresol red color change or quantitatively via EvaGreen fluorescence. Fluorescent and colorimetric dual-mode LAMP methods have been reported previously. Fluorescence dyes and neutral red are the most commonly used indicators in current dual-mode LAMP assays. The use of fluorescence dyes alone requires colorimetric observation with the aid of ultraviolet or blue-light. Neutral red interferes with the absorption peaks of fluorescent dyes such as SYBR Green and EvaGreen, and is therefore often used in combination with Texas Red [33]. For example, Khamsingnok et al. established a colorimetric RT-LAMP targeting the ORF2 gene of FCV using neutral red as the indicator, which allowed clear visual interpretation of results (pink for positive and yellow for negative) [34]. Rapichai et al. developed a dual-mode LAMP assay targeting the pol gene of feline leukemia virus using neutral red and Texas Red as indicators [35]. In this study, we innovatively employed cresol red and EvaGreen for the development of a dual-mode LAMP assay. No interference was observed between the two indicators, as cresol red did not affect the absorption peak of EvaGreen. Moreover, neutral red has a color transition range of pH 6.4–8.0 (red to yellow), whereas cresol red exhibits a color transition range of pH 7.2–8.8 (yellow to violet). Cresol red is more sensitive in detecting pH shifts during the LAMP reaction, and the color change from violet to yellow is more easily distinguishable by the naked eye.

The developed LAMP assay demonstrated high specificity, showing no cross-reactivity with other common feline URTD pathogens (FCV, *C. felis*, *M. felis*, *Bb* and *Sp*), other feline pathogens (FPV and FCoV), and related herpesviruses (CHV-1 and PRV). The LOD was 10 copies by fluorescence and 100 copies by visual colorimetric observation. In previous research, the LOD of colorimetric LAMP ranged between 10 and 100 copies, such as 1.7 × 10^1^ copies/µL for FCoV [36], 14.3 × 10^1^ copies/µL for FCV [34], 264 copies/μL for ASFV [37], and 50 copies/μL for PEDV [38], etc., which is consistent with the LOD of the LAMP assay developed in this study. The assay also showed excellent reproducibility, producing stable results for strongly, moderately, and weakly positive plasmid samples.

Clinical performance was evaluated using 87 nasal swabs from cats with suspected URTD, with qPCR as the reference method. The LAMP assay detected 44 positive samples, achieving 100% specificity and 95.7% sensitivity compared to qPCR. The overall agreement between the two methods was 97.7% with a kappa value of 0.95, indicating almost perfect agreement. The 95% CI for specificity was 89.9–100.0%. This relatively narrow CI with a lower bound of 89.9% indicates that the LAMP assay possesses excellent ability to rule out negative results, making it suitable as a rapid POCT tool. In contrast, the 95% CI for sensitivity (82.9–99.2%) was significantly wider than that for specificity, primarily due to the relatively small sample size of this study, which resulted in greater sampling error in the estimation of sensitivity. Furthermore, the lower confidence limit of 82.9% suggests that this method still carries a certain risk of false-negative results. Stratified sensitivity analysis based on Ct values of qPCR (Table S3) revealed the sensitivity was 100% for strongly positive (Ct ≤ 25.0) and moderately positive (25.0 < Ct ≤ 30.0) samples, but decreased to 86.67% for weakly positive samples (Ct ≥ 30.0). No statistical significance was observed in the Cochran–Armitage test (*p* = 0.097). Notably, the two samples that tested negative by LAMP among the weakly positive samples all had Ct values exceeding 35.0 (Table S2). Given the limited number of weakly positive samples, especially those with Ct > 35.0, larger studies are needed for validation. Our results indicate that the established LAMP method has lower sensitivity for weakly positive samples, particularly those with Ct > 35.0. Therefore, for samples with high clinical suspicion but negative LAMP test results, confirmation using alternative diagnostic methods is recommended.

A major advantage of this closed-tube LAMP is the prevention of aerosol contamination, a common cause of false positives in LAMP assays. All detection reagents are added before amplification, eliminating the need for post-reaction tube opening. Additionally, during the experiment, we prepared a primer premix (Solution A) and an enzyme reaction mixture (Solution B), and evaluated the storage stability and testing performance. The results showed no significant loss of testing performance after storage at −20 °C for up to 6 months, simplifying operation and enhancing suitability for POCT. This study has some limitations. First, substances such as EDTA, citrate, and SDS introduced during sample collection and nucleic acid extraction can competitively bind to Mg^2+^, thereby inhibiting the LAMP reaction and leading to false-negative results. Therefore, it is necessary to establish an internal amplification control by adding an internal reference gene prior to nucleic acid extraction. By comparing the detection results of the internal control, the accuracy of the detection method can be significantly improved. Additionally, the pH value of the elution buffer used in the nucleic acid extraction may affect the colorimetric results, necessitating strict control of the pH value of the elution buffer. Second, the assay’s sensitivity for samples with very low viral loads (Ct > 35.0) is slightly lower than qPCR. Future research could incorporate CRISPR-Cas detection to enhance sensitivity. Third, the current assay can only detect FHV-1, while clinical feline URTD cases often involve multiple pathogens. Development of a multiplex LAMP assay for simultaneous detection of FHV-1, FCV, *C. felis* and *M. felis* would further enhance its clinical utility.

## 5. Conclusions

In conclusion, a closed one-tube colorimetric and fluorescent LAMP assay using cresol red as a colorimetric indicator and EvaGreen as a fluorescent dye was successfully developed for the detection of FHV-1 in this study. This LAMP assay not only enables visual detection through the color change from violet to yellow, but also allows for real-time quantitative detection through the fluorescence amplification curve. As a simple, efficient visual detection method with high specificity, good repeatability, and sensitivity nearly identical to that of qPCR, the LAMP assay is more suitable for point-of-care testing of FHV-1 in primary veterinary clinics and field settings. This study provides a novel and reliable detection method for the clinical diagnosis and epidemiological investigation of FHV-1.

## Figures and Tables

**Table 1:** Oligonucleotide sequences of primers used in this study.

Methods	Primer Name	Primer Sequences (5′-3′)	Position ^1^	Amplicon Size (bp)	Reference
LAMP	*_Herp_*F3	GCAATGTATATATGATGTTGGTACA	623–647	209	This study
	*_Herp_*B3	TCCATTTTGGTCGGAGAG	814–831		
	*_Herp_*FIP (F1c-F2)	TCCGTTCATAGTGGGAGAAAAT- TTTTTAACAAAAAATACTAGTTGGCG	706–727 658–683		
	*_Herp_*BIP (B1c-B2)	TAGGCTCGTGGAAACTACAATAGT- CTTTGAAAACACTGAATAATGTGTC	729–752 781–805		
	*_Herp_*LF	GCTTCCCCCACCCATCA	684–700		
	*_Herp_*LB	TTCCGATTCGACGGAGTCA	753–771		
PCR	TK-F	ATGGCGAGTGGAACCATCC	1–19	1032	This study
	TK-R	TTAATGGTATATCGTCAAGGCTTC	1009–1032		
qPCR	FHV-F	ATTTGCCGCACCATACCT	300–317	140	[14]
	FHV-R	GCGAGTGGGAAACAGACC	423–440		
	FHV-P	FAM-CTTTTACATTCCAGACTATCCACAATAACAGG-BHQ-1	319–350		

^1^ Position numbers are determined based on the complete TK gene of FHV-1 C-27 strain (GenBank accession no. FJ478159).

**Table 2:** Intra- and inter-assay repeatability for the developed LAMP assay based on fluorescence amplification curve.

Concentration of Plasmid pMD-TK (Copies/μL)	Intra-Assay (Tp Values)			Inter-Assay (Tp Values)		
	Mean	SD	CV (%)	Mean	SD	CV (%)
10^6^	7.44	0.089	1.21	7.62	0.215	2.81
10^4^	12.65	0.142	1.12	12.25	0.312	2.55
10^2^	19.81	0.272	1.37	20.54	0.775	3.77

**Table 3:** Comparative analysis of LAMP assay and qPCR for detecting FHV-1 from nasal swabs.

LAMP	qPCR			Sensitivity (%) (95% CI)	Specificity (%) (95% CI)	k Value	Accuracy (%)
	ositive	Negative	Total				
Positive	44	0	44	95.7 (82.9–99.2)	100.0 (89.9–100.0)	0.95	97.7
Negative	2	41	43				
Total	46	41	87				

## Data Availability

The data presented in this study are available within the article. Raw data supporting this study are available from the corresponding author.

## Supplementary Materials

The following supporting information can be downloaded at: https://www.mdpi.com/article/10.3390/ani16111651/s1, Figure S1: Construction of pMD-TK Standard Plasmid; Figure S2: Electrophoretic analysis of the LAMP products using different primer sets; Figure S3: Electrophoretic analysis of the products from specificity test of the LAMP assay; Figure S4: Electrophoretic analysis of the products from sensitivity test of the LAMP assay; Table S1: Oligonucleotide sequences of LAMP primer sets designed in this study; Table S2: Detection results of FHV-1 in nasal swabs collected from suspected feline URTD cases using the developed LAMP assay and reference qPCR method. Table S3: Stratified sensitivity analysis based on Ct values of qPCR.

## Abbreviations

The following abbreviations are used in this manuscript:

*Bb*	*Bordetella bronchiseptica*
*C. felis*	*Chlamydia felis*
CHV-1	Canine herpesvirus 1
Ct	Cycle threshold
FHV-1	Feline herpesvirus 1
FCV	Feline calicivirus
FCoV	Feline coronavirus
FPV	Feline panleukopenia virus
LAMP	Loop-mediated isothermal amplification
LOD	Limit of detection
*M. felis*	*Mycoplasma felis*
PCR	Polymerase chain reaction
POCT	Point-of-care testing
PRV	Pseudorabies virus
qPCR	Quantitative PCR
*Sp*	*Streptococcus pneumoniae*
TK	Thymidine kinase
Tp	Time to positivity
URTD	Upper respiratory tract disease
