# Supplementary material for: Development of a Closed One-Tube Colorimetric and Fluorescent LAMP Assay for the Rapid Detection of Feline Herpesvirus 1
Source: Animals (Basel). 2026 May 28;16(11):1651. doi: 10.3390/ani16111651 (PMC13255912; doi:10.3390/ani16111651)
Supplement: Supplementary file 1 [file animals-16-01651-s001.zip › animals-4305623-supplementary.pdf]

animals

MDPI

Article

Development of a Closed One-tube Colorimetric and Fluorescent LAMP Assay for the Rapid Visual Detection of Feline Herpesvirus 1

Shushuai Yi[1,2], Han Zhao[1], Xinying Huang[1], Jiaxin Yu[1], Wanli Sha[1], Wei Lian[2], Jiangting Niu[1,2*], Baishuang Yin[1*]

[1] College of Animal Science and Technology, Jilin Agricultural Science and Technology College, Jilin 132101, China; yishushuai888@jlnku.edu.cn (S.Y.); zhaohan4566@163.com (H.Z.); huangxinyingsyx@163.com (X.H.); Mrsuanwork@163.com (J.Y.); jlshawanli@163.com (W.S.); niujiangting888@jlnku.edu.cn (J.N.); yinbaishuang@jlnku.edu.cn (B.Y.)

[2] Jilin Zhengye Biological Products Co.,Ltd, Jilin 132101, China; ZYyanfa123@163.com (W.L.)

* Correspondence: niujiangting888@jlnku.edu.cn (J.N.); yinbaishuang@jlnku.edu.cn (B.Y.)

Supplementary Materials

Table S1. Oligonucleotide sequences of LAMP primer sets designed in this study.

Primer set	Primer name	Primer sequences (5′-3′)	Position[1]
Set 1	$_{Herp}$F3-1	GTCCGCATTTACATAGATGG	61-80
	$_{Herp}$B3-1	AGCTGATAATTCACCACGT	243-261
	$_{Herp}$FIP-1	GGTCGATTTTCATCCGCTCTGA-	122-143
	(1F1c-1F2)	GCCTATGGAATAGGTAAGAGTT	82-103
	$_{Herp}$BIP-1	ACTTACTACTTCCCAGAACCAATGC-	151-175
	(1B1c-1B2)	GGCATAGATACCACCGAC	208-225
	$_{Herp}$LF-1	CTGACCAGGTACTTCGCCGTT	105-125
	$_{Herp}$LB-1	TACTGGCGTAGTCTCTTTGAAACTG	178-202
Set 2	$_{Herp}$F3-2	GTCCGCATTTACATAGATGG	61-80
	$_{Herp}$B3-2	CGGTGATATAGGCAGCATC	265-283
	$_{Herp}$FIP-2	GTAAGTATATCCCGGTCGATTTTC-	133-156
	(2F1c-2F2)	ATAGGTAAGAGTTTAACGGCG	91-111
	$_{Herp}$BIP-2	GGCGTAGTCTCTTTGAAACTGAT-	182-204
	(2B1c-2B2)	TCAGCTGATAATTCACCACG	244-263
	$_{Herp}$LF-2	CCGCTCTGACCAGGTACTT	112-130
	$_{Herp}$LB-2	GTCCAGGACCGGAAACGA	226-243
Set 3	$_{Herp}$F3-3	ACCAATGCTATACTGGCG	168-185
	$_{Herp}$B3-3	ACTTTCTGATATCCTGTTATTGTG	339-362
	$_{Herp}$FIP-3	TGATAATTCACCACGTCGTTTCC-	236-258
	(3F1c-3F2)	TAGTCTCTTTGAAACTGATGTTGT	186-209
	$_{Herp}$BIP-3	GCTGAAGATGCTGCCTATATCAC-	259-281
	(3B1c-3B2)	GATAGTCTGGAATGTAAAAGAAGG	315-338
	$_{Herp}$LF-3	GGTCCTGGACGGCATAGATACCAC	212-235
	$_{Herp}$LB-3	ACTATCAAGCAAGATTTGCCGCA	287-309
Set 4	$_{Herp}$F3-4	GCAATGTATATATGATGTTGGTACA	623-647
	$_{Herp}$B3-4	TCCATTTTGGTCGGAGAG	814-831
	$_{Herp}$FIP-4	TCCGTTCATAGTGGGAGAAAAT-	706-727
	(4F1c-4F2)	TTTTTAACAAAAAATACTAGTTGGCG	658-683
	$_{Herp}$BIP-4	TAGGCTCGTGGAAACTACAATAGT-	729-752
	(4B1c-4B2)	CTTTGAAAACACTGAATAATGTGTC	781-805
	$_{Herp}$LF-4	GCTTCCCCCACCCATCA	684-700
	$_{Herp}$LB-4	TTCCGATTCGACGGAGTCA	753-771

[1]Position numbers are determined based on the complete TK gene of FHV-1 C-27 strain (GenBank accession number: FJ478159).

Table S2. Detection results of FHV-1 in nasal swabs collected from suspected Feline URTD cases using the developed LAMP assay and reference qPCR method. The time to positivity (Tp) values based on fluorescent LAMP assay and cycle threshold (Ct) values based on qPCR were listed in table. Violet shading indicated a negative reaction by colorimetric LAMP, whereas yellow shading indicated a positive reaction; light blue shading indicated a positive qPCR result.

**Sample ID**	24A01	24A02	24A03	24A04	24A05	24A06	24A07	24A08	24A09	24A10
**LAMP**	Tp=12.73	Tp=11.88	N	Tp=14.63	N	N	N	Tp=23.42	Tp=8.57	N
**qPCR**	Ct=26.55	Ct=23.18	N	Ct=27.18	N	N	N	Ct=33.50	Ct=20.98	N
**Sample ID**	24A11	24A12	24A13	24A14	24A15	24B01	24B02	24B03	24B04	24B05
**LAMP**	Tp=11.98	N	Tp=9.03	Tp=15.03	N	Tp=23.91	Tp=20.75	N	Tp=11.88	Tp=8.03
**qPCR**	Ct=25.32	N	Ct=21.64	Ct=27.46	N	Ct=33.82	Ct=30.25	N	Ct=25.55	Ct=20.08
**Sample ID**	24B06	24B07	24B08	24B09	24B10	24B11	24C01	24C02	24C03	24C04
**LAMP**	N	Tp=11.75	Tp=23.57	N	N	Tp=24.15	Tp=13.78	N	N	Tp=10.05
**qPCR**	N	Ct=25.30	Ct=33.28	N	Ct=35.75	Ct=33.25	Ct=26.19	N	N	Ct=22.20
**Sample ID**	24C05	24C06	24C07	24C08	24C09	24C10	24C11	24C12	24C13	24C14
**LAMP**	Tp=14.19	N	N	Tp=11.52	Tp=20.95	N	Tp=11.71	N	Tp=15.73	Tp=16.02
**qPCR**	Ct=27.85	N	N	Ct=25.75	Ct=30.33	N	Ct=25.57	N	Ct=28.85	Ct=28.87
**Sample ID**	24D01	24D02	24D03	24D04	24D05	24D06	24D07	24D08	24D09	24D10
**LAMP**	Tp=20.27	N	Tp=19.58	Tp=19.75	N	Tp=8.57	N	Tp=8.99	N	N
**qPCR**	Ct=30.55	N	Ct=29.81	Ct=30.50	N	Ct=20.16	N	Ct=21.73	N	N
**Sample ID**	25A01	25A02	25A03	25A04	25A05	25A06	25A07	25A08	25A09	25A10
**LAMP**	N	Tp=12.31	N	N	Tp=19.83	N	Tp=20.19	N	Tp=13.56	Tp=13.71
**qPCR**	N	Ct=26.33	Ct=36.79	N	Ct=29.57	N	Ct=30.55	N	Ct=27.93	Ct=27.81
**Sample ID**	25A11	25A12	25A13	25A14	25A15	25A16	25A17	25A18	25B01	25B02
**LAMP**	N	N	N	Tp=7.85	Tp=8.90	N	Tp=24.01	N	N	N
**qPCR**	N	N	N	Ct=20.05	Ct=21.53	N	Ct=33.73	N	N	N
**Sample ID**	25B03	25B04	25B05	25B06	25B07	25B08	25B09	25B10	25B11	25B12
**LAMP**	N	N	Tp=20.87	N	Tp=10.50	Tp=12.05	N	Tp=12.83	N	N
**qPCR**	N	N	Ct=30.54	N	Ct=22.22	Ct=25.36	N	Ct=25.71	N	N
**Sample ID**	25B13	25C01	25C02	25C03	25C04	25C05	25C06			
**LAMP**	Tp=24.05	Tp=22.98	N	Tp=14.05	N	Tp=11.15	N			
**qPCR**	Ct=33.91	Ct=32.77	N	Ct=27.10	N	Ct=23.98	N			

Table S3. Stratified sensitivity analysis based on Ct values of qPCR.

**Ct value**	**Positive for qPCR**	**Positive for LAMP**	**Sensitivity (%)**	**95% confidence interval (CI)**
Ct≤25.0	11	11	100	67.86~100
25.0＜Ct≤30.	20	20	100	79.95~100
Ct≥30.0	15	13	86.67	58.39~97.66

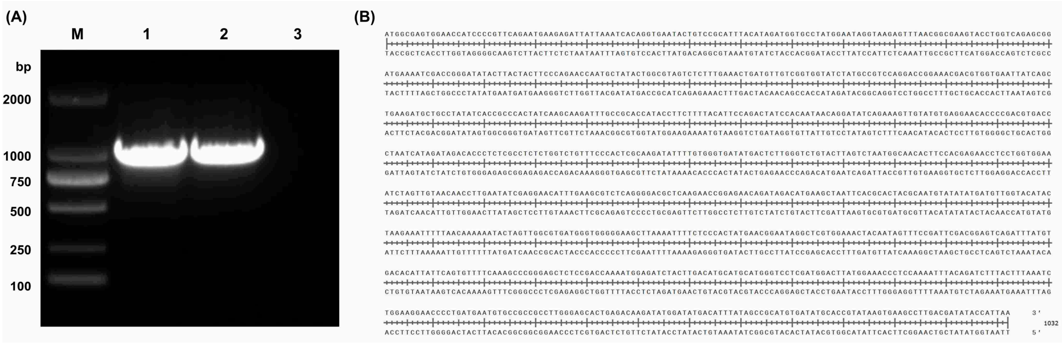

Figure S1. Construction of pMD-TK Standard Plasmid. (A) PCR amplification results of the FHV-1 TK gene. Lane M, DL2,000 DNA Maker (TAKARA, China); Lane 1~2, FHV-1 isolate CH-B; Lane 3, negative control. (B) Sequence information of the plasmid pMD-TK.

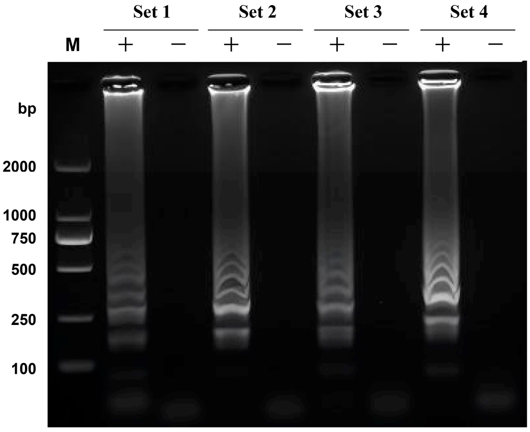

Figure S2. Electrophoretic analysis of the LAMP products using different primer sets. Lane M, DL2,000 DNA Maker (TAKARA, China); Set 1-4, LAMP products using primer set 1-4; + indicated that viral DNA extracted from FHV-1 isolate CH-B was used as template for LAMP amplification. − indicated the negative control.

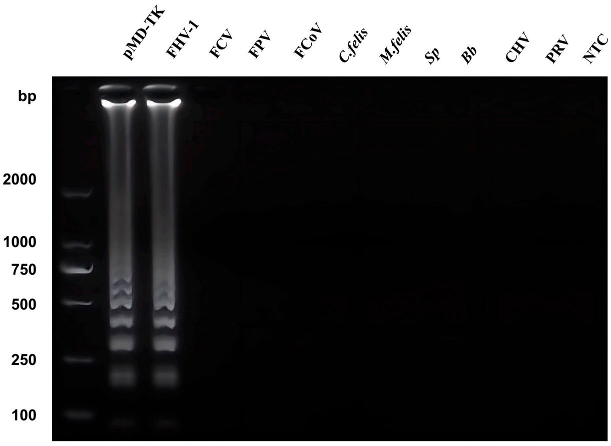

Figure S3. Electrophoretic analysis of the products from specificity test of the LAMP Assay.

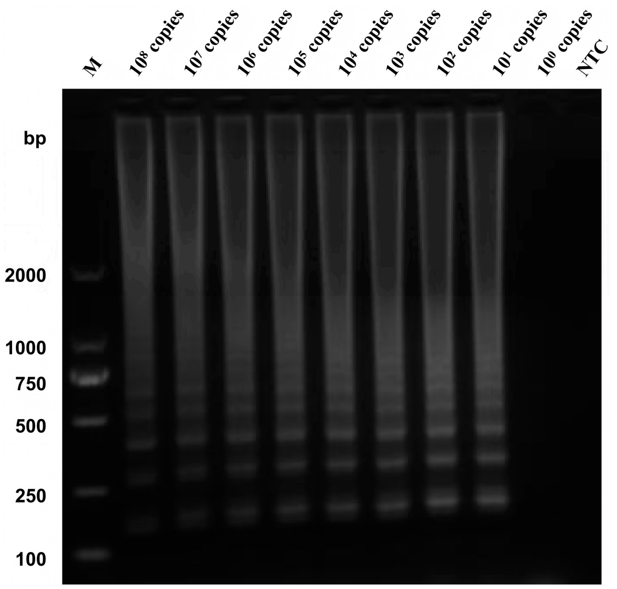

Figure S4. Electrophoretic analysis of the products from sensitivity test of the LAMP Assay.
